# Practical and ethical complexities of MAiD: Examples from Quebec

**DOI:** 10.12688/wellcomeopenres.16306.1

**Published:** 2020-10-01

**Authors:** Gitte Koksvik

**Affiliations:** 1 Department of Philosophy and Religious Studies, Programme for Applied Ethics, Norwegian University of Science and Technology, Trondheim, Norway; 2 End of Life Studies Group, School of Interdisciplinary Studies, University of Glasgow, Dumfries, UK

**Keywords:** Assisted dying, Canada, Conscientious objection, Ethics, Euthanasia, MAiD, Medical Aid In Dying, Quebec

## Abstract

**Background: **Legally practiced assisted dying is an ethically complex area in need of empirical and conceptual work. International research suggests that providing assisted dying may be experienced as rewarding and meaningful but also emotionally and psychologically taxing, associated with feelings of loss and loneliness. Yet little research has been published to date, which attends to the long-term effects of providing assisted dying. In this article, I contribute to filling this gap in the literature using the Canadian province Quebec as an illustrative case. Medical aid in dying (MAiD) in the form of physician provided euthanasia has been a lawful end of life healthcare option in Quebec since December 2015 and significant research is currently emerging from this jurisdiction.

**Methods: **In this article, I draw on nine in-depth interviews with Quebec physicians, all of whom engaged with end of life care in different ways.

**Results: **Four of the interviewed physicians provided medical aid in dying (MAiD) and five did not. The major themes of MAiD in relation to aggressive treatment, conscientious objection and uneven distribution of work emerge, and it appeared clearly that MAiD was experienced and thought of as qualitatively different to other end of life procedures.

**Conclusions: **Our findings expose a complexity and contentiousness within the practice, which remains under researched and underreported and indicate avenues where more research is needed.

## Introduction

Although controversial and contested, right to die advocacy is gaining traction worldwide and more jurisdictions are passing laws to allow a form of assisted dying. In Europe and North America, it has become an integral part of the ways in which people think about dying ( Richards & Krawczyk, 2019). It is surprising therefore, to find few studies dealing with the effects of the practice on health professionals or about the practicalities of being a provider of assisted death ( Khoshnood *et al*., 2018). International publications, which speak to the psychological or emotional aspects of providing assisted dying, indicate that this may be experienced as a very meaningful practice, yet one that it is also deeply taxing ( Evenblij *et al.,* 2019; Georges *et al.,* 2008; Haverkate *et al*., 2001; Shaw *et al*., 2018; van Marwijk *et al.,* 2007; Voorhees *et al.,* 2014).

In the following, I aim to make a contribution to fill this gap in the literature, using the Canadian province of Quebec as an illustrative case. Quebec legalised assisted dying in the form of physician-administered euthanasia as part of healthcare in December 2015 and research is currently emerging in earnest from the province. I draw upon evidence in the published literature and supplement it with empirical findings from in-depth semi-structured interviews with nine Quebec physicians. Surely, nine interviews are not enough to draw generalizable conclusions about the experiences of physicians in Quebec as a whole. Nevertheless, these rich accounts indicate a complexity and contentiousness within the practice, which remains under researched and underreported. It is not my intention in this article to reflect on the ethical justification of assisted dying, and I argue neither for nor against it.

## Methods

### Ethical statement

Our study obtained ethical clearance from the Research Ethics Committee of the University of Glasgow, College of Social Sciences (Application No. 400180010) and interviewees received detailed information about the study beforehand and provided written informed consent to participate.

### Study design

The empirical materials used in this paper stem from a larger qualitative interview study with 29 professionals involved with palliative care and/or assisted dying in Flanders (Belgium), Oregon (USA) and Quebec (Canada). The objective of this study was to explore the relationship of palliative care with assisted dying in these settings, from the perspective of palliative care clinicians and other professionals involved in both assisted dying and palliative care, going beyond the official statements about anticipated or feared impacts of assisted dying legalisation to learn about how this unfolds in practice. More detail on this study, methods and the recruitment process is described elsewhere ( Gerson *et al*., 2020a; Gerson *et al*., 2020b).

### Participant selection

We undertook purposive sampling to recruit professionals in each jurisdiction with experience working with palliative care and/or assisted dying. Participants were identified through extensive internet and literature searches, professional networks and snowballing. Participants were interviewed as individuals and not as representatives of their respective organizations, institutions or workplaces.

### Data collection

We constructed an interview schedule based on the objectives of the study and on points of contention identified in the literature ( Gerson *et al*., 2019a). Interviewees were asked about: their experiences working either in palliative care, with assisted dying or both; whether they had experienced or knew of differences in the field or practice of palliative care following the legalization of assisted dying; about the nature of the relationship between the two; their impressions of the general public’s knowledge and attitudes; and about the challenges or benefits brought to palliative care by assisted dying. In each interview, moreover, the participants were encouraged to speak freely on the broad themes of the study in order to emphasise and express what was of the greatest interest to them. 

Another researcher in the main project and I, both research associates at the time and experienced in qualitative interviewing, conducted all the interviews in the main study. The interviews lasted 1–2 hours and were audio recorded. Six of the interviews took place face-to-face, the remaining three were conducted via Skype and telephone. They were transcribed verbatim - and in the case of three conducted in French translated, by a professional agency.

### Data analysis

To ensure trustworthiness, the two interviewers each independently analysed the transcripts, employing an iterative and thematic approach, both using manual coding in the programme NVIVO 12. The findings were subsequently discussed within the research team. I subsequently undertook a more in-depth reading of the Quebec transcripts, building on the initial analysis. The interpretation of the data was inductive and exploratory in character. I render quotes to give a sample of the richness of the responses and to favour the voices of the respondents. The language has been altered only to facilitate anonymisation and to remove grammatical errors and delete repeated words.

## MAiD in Canada and Quebec

Medical aid in dying (MAiD) ( *Aide médicale à mourir*, AMM) was legalised in Quebec as part of healthcare provision in December 2015 ( Act respecting end-of-life care, 2019). This meant that individuals who fulfilled certain criteria could lawfully choose to end their life by receiving lethal medications administered by a physician, a practice also known as euthanasia. Eligibility for MAiD is restricted to patients at the end of life whose deaths are deemed ‘reasonably foreseeable’ and who are experiencing intolerable suffering which cannot be alleviated in a way that the individual finds acceptable. The impact of MAiD legislation in Quebec has been significant and the number of requests quickly exceeded expectations ( Ummel & Vachon, 2017). In 2017, MAiD accounted for 1.09% of deaths in the province. This number is only slightly lower than the figures in Belgium, where assisted dying has been lawful since 2002. Moreover, in Quebec, the demand appears to be growing; MAiD increased by 73% between January 2016 and March 2018 ( Commission sur les soins de fin de vie, 2019) and the situation is evolving quickly. In September 2019, a Quebec judge ruled that the criteria in the law limiting MAiD to patients at end of life were unconstitutional ( Ha & Grant, 2019). Considering this, it seems likely that the character of MAiD will change in Quebec and possibly in the whole of Canada in the near future.

The Quebec government and medical professional associations have advocated an approach according to which MAiD is an exceptional intervention offered to patients when therapeutic, curative and palliative approaches are deemed unsatisfactory. Yet evidence indicates that this might not fit with the picture on the ground. Indeed, little is known about how MAiD requests are approached or how they fit in the broader context of end of life care (Seller & Bouthillier, 2019). Moreover, a large proportion of doctors have refused to practice it ( Selby *et al.,* 2020). Until recently, research about MAiD largely focused on the understanding of end-of-life legislation, cost analysis, and program implementation ( Li *et al.,* 2017; Marcoux *et al.,* 2015; Trachtenberg & Manns 2017; Wales *et al.,* 2018). However, research is currently emerging which looks in more detail at the experiences of physicians and nurses. This research presents a substantively positive image of MAiD provision, although problems regarding institutional disagreements, time consumed and financial issues have been raised ( Brooks, 2019; Bruce & Beuthin, 2019; Heilman & Trothen, 2019; Khoshnood *et al.,* 2018; McKee & Sellick, 2018).

## Results

Nine interviews with physicians in the Quebec region were carried out in Spring 2019 – four women and five men. Throughout the text, I use the gender neutral ‘they’ to ensure anonymity and the respondents are identified by number only. The interviewees had between 10 and 30 years of experience and worked in different fields of medicine including general practice, intensive care, community medicine and palliative care. Some worked in hospitals, others in clinics, education, and hospice. Several participants had more than one appointment and place of work. Six were recruited directly, three through snowballing.

### Medical aid in dying and excessive treatment

Four of the physicians interviewed in our study provided MAiD as part of their professional activities, whereas five did not. Accordingly, MAiD proved a divisive topic. However, both interviewees who did and interviewees who did not practice MAiD situated it in relation to a wider culture of medical interventionism and excessive treatment ( *acharnement thérapeutique, Fr*). Indeed, concerns emerged across the interviews about excessive, futile treatment being the norm. Some shared the view that the advocacy preceding MAiD legalisation had painted an image of only two possible dying trajectories, whereby MAiD became synonymous with a good death and a non-MAiD death was described as one *necessarily* plagued by excessive treatment, protracted pain and indignity. This rhetoric was pronounced in the Carter *v* Canada proceedings leading up to legalisation ( Broom 2016; Karsoho *et al*., 2016). Yet, the relationship between aggressive and life-prolonging treatments and MAiD is not straightforward. Seller *et al*. (2019) recently found that out of 80 patients who requested MAiD, 29% would still have received potentially lifesaving or life prolonging interventions in case of emergency, including in most cases, resuscitation. While 86% of patients consulted palliative care, the remaining 14% did not, most often because the patient refused to do so. Several of our interviewees described similar situations, which in their view complicated the issue of MAiD because it affected delivery as well as how the wider population views its options:

This I found quite frustrating and illogical (…) There is over-treatment and aggressive treatment and we don’t try to address this problem, but still we offer voluntary end of life; I find it quite irresponsible. If we worked better at the source (…) If we address the question, if we talk about dying (…) then yes, okay, voluntary dying [will exist], but at least it wouldn’t be a solution [to] being fed up after having endured so much cruelty beforehand (Physician 5).

They are pushing active care right to the boundaries of death. (…) I would say that’s (…) indirectly related to MAiD in the sense that things become more abrupt. (…) So, you’re going from chemo on Monday to MAiD on Tuesday, almost. Windows become very compressed, you don’t have the gradual decline that you had in the past and that’s making it hard to time MAiD, when to recommend it, and I think patients’ expectations of treatments are higher (Physician 2).

In light of this, some thought the main issue was not the legality of MAiD, but the medical system itself. At the same time, however, and quite paradoxically, there was consistent description of patients who request MAiD as possessing a particular character and of being more focussed on control and self-determination than the general population. This is supported by the international literature, which indicates that the desire for MAiD reflects an individual’s long-standing beliefs about life and death, more so than their care options ( Hendry *et al.,* 2012) and where, indeed, control is identified as an important motivation for, and even experienced outcome of, MAiD being a clinical possibility (Ball *et al*., 2019; Seller *et al.,* 2018; Wiebe *et al*., 2018)

### Practice and provision: The uneven burden of distribution

The physicians who provided MAiD were generally happy with their practice, although a couple had experienced problems due to disagreements with institution management or other professionals. They felt satisfaction at being able to help patients achieve a good death in line with their priorities and values. A death, moreover, which these physicians believed would lead to less cumbersome bereavement for next of kin. Contrary to ‘natural death’, MAiD was understood to allow patients the choice of place, getting their affairs in order and allowing for planning a choreographed farewell, as coined by Buchbinder (2018). They expressed receiving a lot of gratitude from both patients and their families, and in line with the findings of Bruce & Beuthin (2019), deaths resulting from the provision of MAiD were more than once described as ‘beautiful’. Nevertheless, these interviewees did not present an unequivocally positive image of MAiD. Instead, they raised concerns, which warrant further attention.

Because MAiD is part of healthcare provision in Quebec, public institutions and hospitals are obligated to offer it on their premises and physicians of all specialties and areas of practice can evaluate and provide MAiD. In the words of one: ‘When it was passed, what they thought was that every doctor would take care of their own patients’ (Physician 3). However, despite the wide public support for the change in law and the increasing number of patients seeking to avail themselves of it, it appears that MAiD is not evenly distributed, either among physicians or health institutions ( Quinn & Detsky, 2017; Schiller, 2017). This was reflected by our interviewees as well:

I can’t wait for the day when the burden - of time, resources and on the psychological level - will be redistributed on a broader spectrum of care. It would really be advisable (Physician 8).

Clinicians have the right to object to evaluating patient requests and to providing the procedure itself on the grounds of conscience but are legally required to make referrals in the case of a request. Indeed, it appears that many physicians have chosen to abstain from the practice of MAiD ( Heilman & Trothen, 2019). Interviewees who provided MAiD lamented that there were very few of them in the province, and even fewer yet who agreed to perform MAiD outside of the hospital setting such as in the patient’s home. Several of our interviewees who worked full or part time in hospitals reported that very few or even no physicians in their institution provided MAiD. In hospitals or units where no MAiD providers work, calls have to be made to other units or institutions to bring in physicians to complete the task, an arrangement described by one as ‘a puzzle every time’, since this requires changing work to accommodate abstention within the care team. Moreover, provision would seem vulnerable to illness, burnout or retirement within a team of practitioners.

Such arrangements may, at the very least, infringe upon the notion of MAiD as one of several possible, natural conclusions to an ongoing physician-patient relationship. For providing physicians too, this was problematic. One expressed that if they were going to be off work for a while or go on holiday, this put both them and their patients in a difficult position. For the patient, this would mean either waiting longer before accessing MAiD – thus suffering unbearably and in some cases, running the risk of losing capacity to consent; or scheduling MAiD pre-emptively, by which patients may ‘have to’ die sooner if indeed they wish to access assisted dying. For the physician, this caused feelings of guilt and unease about taking time off from work.

MAiD takes time. The interviewees described strict rules regarding the medications and the kits in which it comes, which must be returned to the hospital pharmacy after MAiD is performed. If MAiD is performed outside the hospital therefore, and in rural or remote areas especially, this involves a lot of driving and is not always well remunerated. The evaluation process itself also takes time and because MAiD is understood to be patient-led, physicians, it seemed, often significantly adapted their schedules:

Usually when people ask for MAiD, they [want it] in the evenings or the weekends because they want the family to be there. It’s a big change because (…) usually it involves the whole family unit and the friends. (…) You have 2–3 meetings with the family and the patient and the friends sometimes before the MAiD provision. It takes a lot of time (Physician 6).

Because provincial and federal laws are different, Quebec physicians described having to fill out to sets of documentation in each instance of evaluating and performing MAiD. This was unanimously described as lengthy and tedious:

The main problem we have is with the declaration. We have to fill in an 11-page formulary after every single MAiD provision and also those who are refused (Physician 2)

Altogether, this means that provision of MAiD can be experienced as ‘a big, big hassle’ (Physician 3). Indeed, some were sure that inconvenience rather than conviction was at the root of many physicians not getting involved:

There is practical resistance for a portion of the doctors because it’s complicated. It’s time-consuming too, so people are just saying ‘I don’t have time to do this, I cannot add this to my schedule’ (Physician 4).

### Not like other interventions

As previous research has underlined, assisted dying may not only be taxing practically but can be experienced as a contentious task emotionally or psychologically, associated with heavy, traumatic or difficult responsibilities, loneliness, mixed feelings and even a sense of loss ( Wales *et al*., 2018). This was evident in our materials as well. One physician stated being ‘relatively at ease’ with their cases, elaborating that ‘each one becomes easier and easier’:

I didn’t like it at first. I still don’t love it but I don’t want to tell patients that. It was a funny feeling the first time, I felt really weird. I went back to the office and I was having butterflies. I’m not an anxious person, I’ve been involved in lots of stuff, I’ve seen lots of people die and I felt weird. I felt, ‘gees, I’ve just killed somebody’ (Physician 2).

Another described a ‘shock’ reaction to taking up MAiD provision, especially in cases where the patient did not look imminently dying and where the transition from life to death appeared abrupt. Oftentimes, they explained, nurses would cry afterwards, because MAiD signified professionals ‘losing their bearings’ (Physician 6). The difficulty of this was heightened by the discrepancy between the availability of providing physicians and patient demands. One interviewee had conducted more than 20 procedures of MAiD per month over several consecutive months. Another described being the only MAiD providing physician in their institution:

That was very heavy because it meant that, you know… it’s terrible… like for instance, recently I had to do 2 interventions of MAiD in 24 hours. That’s too much (Physician 3).

This sentiment was shared by another who said:

I wouldn’t like to do a lot. I wouldn’t be able to do one or two a week, it’s too difficult for me. So, once in a while is okay, to help a patient, but not that much (Physician 4).

Indeed, for our interviewees involved in MAiD it was important to maintain self-enforced limits:

I believe in the ideology 100% but it’s not a comfortable feeling sometimes and I think that may be one reason why I’m sticking to the hard core indications (…) I’m quite conservative (…) I’m not one of those who pushes the frontier, who says, ‘Oh you’re old, you’re tired of living let’s give you MAiD’. I want the hard-core criteria (Physician 2).

One interviewee explained that they were pleased with the way the Quebec law was formulated, because it provided the work with a clear clinical reference. This physician found the ongoing court appeals to extend eligibility criteria for MAiD unacceptable. On the one hand, this appeared a desire to maintain clinical control to discern and determine the process of end of life. There was also however, an implicit wish to avoid professional instrumentalisation and an expressed fear of a *slippery slope* whereby vulnerable individuals would experience a social loss of value:

I don’t want MAiD to become a checklist, technical thing (…) where “yes, yes, yes, you’ve got it” and then any technician can arrive and (…) inject the patient and he’s going to be dead. (…) I have less problem giving meds to euthanize a patient who is *asking me* for euthanasia than holding a person with dementia and shooting an IV, a person who doesn’t know what’s happening (…) This is more like an execution in my point of view (Physician 6).

Another interviewee, however, believed the inclusion criteria were too narrow but that this was part of a process of gradual societal change.

### Conscientious objection

Many physicians across the province have chosen conscientious objection to participating in MAiD. However, despite a few examples to the contrary, interviewees who practiced MAiD expressed that other physicians in their institutions were generally grateful. Their impression was that many non-practicing physicians were happy that *someone* was doing it, so that they themselves did not have to. Some interviewees were not against assisted dying itself but did not want it to be a medical task, arguing instead for assisted suicide or other non-clinical provision options. Supporting the findings of Bouthillier & Opatrny (2019), one interviewee opined that many of their colleagues had nothing against MAiD philosophically, but rather did not feel comfortable doing it, leading them to express guilt and attempting to justify their choice, saying that they were not ‘ready’ but that ‘maybe they should’. Conceivably, there is a professional pressure for physicians (and clinicians generally) to embrace MAiD provision. In the words of one:

I think it’s more complex than just saying we can exercise our right [to object] (…) The right is in the law, it’s clear (…) [But] the way things are, this is not realistic (Physician 9).

Both MAiD providers and non-providers related experiences of pressure from patients and families. Some were accused of not having their patients’ best interest in mind and were told that their objection was a form of activism or a defiant attempt to put obstacles in people’s way:

It’s been very divisive, it’s been very difficult, it’s not easy. People don’t understand. They will very often try to explain this as another expression of medical power (Physician 9).

Expectedly perhaps, declining the MAiD request of a suffering patient was experienced as difficult. Sometimes declining a request could be a source of criticism from both patients and next of kin as well as from other colleagues, due to ambiguities within the criteria themselves. Moreover, there were anecdotal examples of ‘doctor hopping’ where patients whose request had been denied subsequently sought out new physicians until being accepted, which seemed a source of stress to MAiD providers.

## Concluding remarks

Our interviews revealed conflicting views about the ethical permissibility of MAiD. However, they also exposed nuances within these opinions, which did not always come across either as a full rejection or as a wholehearted embrace of the practice. A close reading of the interviews uncovered preoccupation with excessive treatment and medical interventionism, the practicalities of work and the realities of objection. The apparent absence of time, between aggressive life prolonging measures and deliberately causing death, may speak to a medico-social denial of death, by which the period of decline, where there is no longer an objective of cure, appears unacceptable ( Richards & Krawczyk, 2019). It may also be indicative of the effects of financial austerity having contributed to underdeveloped, underfunded and unequally distributed palliative care services ( Ummel & Vachon, 2017). If we grant that aggressive medical treatment can be a source of suffering and pain, liable to be experienced as unbearable if no longer serving life-ameliorating purposes, this may generate demands for MAiD that *might not* have arisen or would arise at a later stage, had a more supportive treatment course been adopted. Indeed, for some, the practice of aggressive treatment until death makes assisted dying stand out as the (only) humane option. For others, it raises practical questions of ethical consequence relating to the evaluation and determining criteria of MAiD eligibility. Importantly, however, Seller *et al.*’s study (2019) raises the question of what MAiD really means to the population when patients requesting it simultaneously have resuscitation orders in place. So too does the consistent description of patients desiring MAiD as possessing a particular independent or controlling character.

These findings indicate that MAiD is not experienced as ethically equivalent to other end of life options, as some seem to suggest ( DeMichelis *et al.,* 2019). Quite the contrary, several of the interviewees who provide MAiD relay opinions and experiences that, although supporting the practice, evidence it as being ethically and emotionally quite challenging. The fact that MAiD is designated as a right within healthcare, together with the high level of physician abstention, is likely to have significant consequences. For patients, this results in unequal access to care, as defined by law. This is documented in rural areas ( Schiller, 2017). Our findings indicate it might be the case in urban areas as well. For physicians, this leads to an increased burden on those agreeing to provide MAiD and puts pressure on those who object. Crucially, it seems reasonable to assume that physicians who perform MAiD, in most cases do not intend for this to become a decisive or even prominent part of their activities, much less intend to become their institution’s ‘euthanasia doctor’. In some cases, however, this would seem to be the consequence. A nuanced discussion about objection seems timely. Moreover, it would not be possible to conclude from our interviews, as Shaw *et al.* (2018) have, that the MAiD legislation should be updated to be more inclusive. Our findings, together with those of Bouthillier & Opatrny (2019), indicate that reluctance to participate in MAiD is not necessarily based on ideological opposition to assisted dying but on practical concerns regarding professional responsibilities and remuneration as well as fears about emotional and psychological consequences of performing intentionally life-ending actions. These elements of assisted dying provision seem largely ignored by the international literature to date.

## Data availability

The author is unable to make all data publicly available because of the terms of data sharing included in the consent process for this study. Furthermore, it is not possible to effectively de-identify participants and their organisations in reporting the interviews conducted for this study. However, access will be granted to the underlying data on a case-by-case basis upon request to the author (Gitte Koksvik; gitte.koksvik@ntnu.no). This will be granted upon request from a researcher for the purposes of further research, providing the requesting researcher is in possession of a protocol that has been approved by an ethics committee and which satisfies the guarantees of anonymity originally given to the research participants.

## References

[ref-1] Bouthillier ME Opatrny LA: Qualitative study of physicians’ conscientious objections to medical aid in dying. *Palliat Med.* 2019;33(9):1212–1220. 10.1177/0269216319861921 31280666

[ref-2] Broom A: The right to medicalization? Invited commentary on Karsoho et al. (2016). *Soc Sci Med.* 2016;173:104–107. 10.1016/j.socscimed.2016.12.001 27940417

[ref-3] Brooks L: Health Care Provider Experiences of and Perspectives on Medical Assistance in Dying: A Scoping Review of Qualitative Studies. *Can J Aging.* 2019;38(3);384–396. 10.1017/S0714980818000600 30626453

[ref-4] Bruce A Beuthin R: Medically Assisted Dying in Canada: “Beautiful Death” Is Transforming Nurses’ Experiences of Suffering. *Can J Nurs Res.* 2019; 844562119856234. 10.1177/0844562119856234 31188639

[ref-5] Buchbinder M: Coreographing death: A Social Phenomenology of Medical Aid‐in‐dying in the United States. *Med Anthropol Q.* 2018;32(4):481–497. 10.1111/maq.12468 30014621

[ref-6] Chapter S-32.0001 Act respecting end-of-life care.2019; Accessed January 8 2020. Reference Source

[ref-7] Commission sur les soins de fin de vie: Rapport sur la situation des soins de fin de vie au Québec. Du 10 Décembre 2015 au 31 mars 2018.Gouvernement de Québec. ISBN (PDF): 978-2-550-83386-4.2019. Reference Source

[ref-8] DeMichelis C Zlotnik SR Rapoport A: Medical assistance in dying at a pediatric hospital. *J Med Ethics.* 2019;45(1):60–7. 10.1136/medethics-2018-104896 30242079

[ref-9] Evenblij K Pasman HRW van Delden JJM: Physicians’ experiences with euthanasia: a cross-sectional survey amongst a random sample of Dutch physicians to explore their concerns, feelings and pressure. *BMC Fam Pract.* 2019;20(1):177. 10.1186/s12875-019-1067-8 31847816 PMC6918628

[ref-10] Georges JJ The AM Onwuteaka-Philipsen BD: Dealing with requests for euthanasia: a qualitative study investigating the experience of general practitioners. *J Med Ethics.* 2008;34(3):150–155. 10.1136/jme.2007.020909 18316454

[ref-11] Gerson SM Koksvik GH Richards N: The Relationship of Palliative Care With Assisted Dying Where Assisted Dying is Lawful: A Systematic Scoping Review of the Literature. *J Pain Symptom Manage.* 2020a;59(6):1287–1303.e1. 10.1016/j.jpainsymman.2019.12.361 31881289 PMC8311295

[ref-12] Gerson SM Koksvik G Richards N: Assisted dying and palliative care in three jurisdictions: Flanders, Oregon, and Quebec. *Ann Palliat Med.*In press.2020b. Reference Source 10.21037/apm-20-632 33302637

[ref-13] Ha TT Grant K: Québec court strikes down restriction to medically assisted dying law calls it unconstitutional. *The Globe and Mail.* 2019; Accessed 9 January 2020. Reference Source

[ref-14] Haverkate I van der Heide A Onwuteaka-Philipsen BD: The emotional impact on physicians of hastening the death of a patient. *Med J Aust.* 2001;175(10):519e522. 11795538 10.5694/j.1326-5377.2001.tb143707.x

[ref-15] Heilman MKD Trothen TJ: Conscientious objection and moral distress: a relational ethics case study of MAiD in Canada. *J Med Ethics.* 2019;46(2):123–127. 10.1136/medethics-2019-105855 31811013

[ref-16] Hendry M Pasterfield D Lewis R: Why do we want the right to die? A systematic review of the international literature on the views of patients, carers and the public on assisted dying. *Palliat Med.* 2012;27(1):13–26. 10.1177/0269216312463623 23128904

[ref-17] Khoshnood N Hopwood MC Lokuge B: Exploring Canadian Physicians’ Experiences Providing Medical Assistance in Dying: A Qualitative Study. *J Pain Symptom Manage.* 2018;56(2):222–229.e1. 10.1016/j.jpainsymman.2018.05.006 29775692

[ref-18] Li M Watt S Escaf M: Medical Assistance in Dying - Implementing a Hospital-Based Program in Canada. *N Engl J Med N.* 2017;376(21):2082–2088. 10.1056/NEJMms1700606 28538128

[ref-19] McKee M Sellick M: Self Care/Burnout Study Results.Final Report, 2 ^nd^Annual Medical Assistance in Dying Conference;2018;51–52.

[ref-20] Marcoux A Arsenault C: Health care professionals' comprehension of the legal status of end-of-life practices in Quebec: study of clinical scenarios. *Can Fam Physician.* 2015;61(4):e196–203. 26052600 PMC4396778

[ref-21] Karsoho H Fisman JR Wright DK: Suffering and medicalization at the end of life: The case of physician-assisted dying. *Soc Sci Med.* 2016;170:188–196. 10.1016/j.socscimed.2016.10.010 27821302

[ref-22] Quinn KL Detsky AS: Medical Assistance in Dying: Our Lessons Learned. *JAMA Intern Med.* 2017;177(9):1251–1252. 10.1001/jamainternmed.2017.2862 28715539

[ref-23] Richards N Krawczyk M: What is the cultural value of dying in an era of assisted dying? *Med Humanit.* 2019; medhum-2018-011621. 10.1136/medhum-2018-011621 31350304

[ref-24] Schiller CJ: Medical assistance in dying in Canada: focus on rural communities. *J Nurse Pract.* 2017;13(9):628–634. 10.1016/j.nurpra.2017.07.017

[ref-25] Selby D Bean S Isenberg-Grzeda E: Medical assistance in dying (MAiD): a descriptive study from a Canadian tertiary care hospital. *Am J Hosp Palliat Care.* 2020;37(1):58–64. 10.1177/1049909119859844 31256607

[ref-26] Seller L Bouthillier ME Fraser V: Situating requests for medical aid in dying within the broader context of end-of-life care: ethical considerations. *J Med Ethics.* 2019;45(2):106–111. 10.1136/medethics-2018-104982 30467196

[ref-27] Shaw J Wiebe E Nuhn A: Providing medical assistance in dying: Practice perspectives. *Can Fam Physician.* 2018;64(9):e394–e399. 30209113 PMC6135115

[ref-28] Trachtenberg AJ Manns B: Cost analysis of medical assistance in dying in Canada. *CMAJ.* 2017;189(3):E101–5. 10.1503/cmaj.160650 28246154 PMC5250515

[ref-29] Ummel D Vachon M: Soins palliatifs et aide médicale à mourir au Québec: Portrait et tensions. *4ème congrès international francophone de soins palliatifs.* 2017;168–169.

[ref-30] van Marwijk H Haverkate I van Royen P: Impact of euthanasia on primary care physicians in The Netherlands. *Palliat Med.* 2007;21(7):609-614. 10.1177/0269216307082475 17942499

[ref-31] Voorhees JR Rietjens JAC van der Heide A: Discussing physician-assisted dying: physicians’ experiences in the United States and The Netherlands. *Gerontologist.* 2014;54(5):808–817. 10.1093/geront/gnt087 24000266

[ref-32] Wales J Iseberg SR Weiger P: Providing Medical Assistance in Dying within a Home Palliative Care Program in Toronto, Canada: An Observational Study of the First Year of Experience. *J Palliat Med.* 2018;21(11):1573–1579. 10.1089/jpm.2018.0175 30095328

