# Supplementary material for: Practical and ethical complexities of MAiD: Examples from Quebec
Source: Wellcome Open Res. 2020 Nov 23;5:227. Originally published 2020 Oct 1. [Version 2] doi: 10.12688/wellcomeopenres.16306.2 (PMC8063540; doi:10.12688/wellcomeopenres.16306.2)
Supplement: Peer review history

# Peer review: Reviewer response for version 2

**DOI:** 10.21956/wellcomeopenres.18114.r42842

**Published:** 2021-04-16

**Authors:** Carlo Leget

**Affiliations:** 1 Department of Care Ethics, University of Humanistic Studies, Utrecht, The Netherlands

This article addresses an underresearched topic of great importance: the experiences of physicians working in a societal context where medical aid in dying is a legal option after a number of requirements have been met. The nature of the research is exploratory: nine physicians have been interviewed and a global picture is painted of the variation in experiences. Of these nine physicians, only four have provided medical aid in dying. One of them has conducted 'more than 20 procedures of MAiD per month over several consecutive months'.

 This paper contributes to scientific knowledge of how clinicians in Canada experience the euthanasia practice in Quebec. From a methodological point of view I think it is well described and explained what has been done, taking care of ethical demands of anonimity. I do, however, have some suggestions and questions after having read this article.

 First, the number of only four physicians having provided medical aid in dying is a very small sample, even for qualitative research. Knowing that one of these four physicians has conducted more than 20 procedures per month over several months suggests that there is an enormous diversity within this small group of respondents. Also of course the other five physicians provide valuable information about the practice in Quebec, but this does not refer to providing medical aid in dying. So the empirical basis is rather narrow here.

 Second, the number of twenty procedures per month over several months is extreme. I do not know any physician in my country (The Netherlands, where medical aid in dying is a legal option since 2002) who comes close to this amount of procedures. Most physicians will have a couple of procedures per year. This raises a lot of questions about the system and the person involved. These questions are not discussed or answered. In that sense the article is better in reporting what is the case then interpreting how to understand the state of affairs.

 Third, at the end of the article I would have expected more reflection and ideas on what the knowledge gained in this research means for further research: what are the next questions to be adressed based on this paper?

Is the work clearly and accurately presented and does it cite the current literature?

Yes

If applicable, is the statistical analysis and its interpretation appropriate?

Not applicable

Are all the source data underlying the results available to ensure full reproducibility?

Yes

Is the study design appropriate and is the work technically sound?

Yes

Are the conclusions drawn adequately supported by the results?

Yes

Are sufficient details of methods and analysis provided to allow replication by others?

Yes

Reviewer Expertise:

Ethics, end-of-life issues, palliative care, spirituality

I confirm that I have read this submission and believe that I have an appropriate level of expertise to confirm that it is of an acceptable scientific standard.

# Peer review: Reviewer response for version 2

**DOI:** 10.21956/wellcomeopenres.18114.r42837

**Published:** 2021-03-05

**Authors:** Scott Y. H. Kim

**Affiliations:** 1 Department of Bioethics, Clinical Center, National Institutes of Health, Bethesda, MD, USA; 2 Department of Psychiatry, University of Michigan Medical School, Ann Arbor, MI, USA

The article reports on a qualitative interview study of 9 Canadian physicians from Quebec regarding their experience with medical aid in dying (MAID). The purposive sampling seems reasonable given the small N that was part of a larger study (as explained by the author), and the analysis thematic, inductive, and exploratory which seems appropriate for what is essentially an exploratory study. There is a small but growing body of literature on the experiences of health professionals who are involved in various forms of MAID, and small sample in depth interview studies such as this do not seem unusual. The author does an excellent job of placing the results in the context of that literature; the reader gains insights about how the concerns and experiences of the physicians in Quebec compare with known issues from other studies. The author does an excellent job of organizing the material thematically, and even-handedly, as the results provide nuance into the experiences of the physicians (e.g., the practical reasons for why some doctors end up performing so many cases, reluctance is not always because of a stance against MAID, belief in MAID is not incompatible with emotional strain in performing, the emergence of peer and family pressures, etc.) rather than ‘taking sides.’ Still, the author raises useful questions in the Concluding remarks in light of the results that are interesting and ethically evocative. Overall, despite its small N, the careful and nuanced reading of the interviews provides useful insights about what it must be like to be a physician encountering MAID requests and practices in Quebec.

 Some minor points. At one point, the author made is sound like only physicians are involved in MAID (e.g., bottom page 5, left column) but in Canada, unlike in other jurisdictions, other health professionals such as nurse practitioners can evaluate requests and perform MAID.

 It was odd to see the section “MAiD in Canada and Quebec” – which seems like background—sandwiched between Methods and Results. Perhaps this is consistent with this journal’s policies, I don’t know. Another somewhat unusual aspect of the MS is that the Results section is essentially an integration of Results and Discussion, perhaps necessitated by the nature of the study—I think it worked fine but it is a bit unusual for someone like me who is used to reading medical journals.

 Finally, I gave 'partly' for q on source data but this is because the author is constrained from providing the full information, as she explains--an explanation that seems quite reasonable to me.

Is the work clearly and accurately presented and does it cite the current literature?

Yes

If applicable, is the statistical analysis and its interpretation appropriate?

Not applicable

Are all the source data underlying the results available to ensure full reproducibility?

Partly

Is the study design appropriate and is the work technically sound?

Yes

Are the conclusions drawn adequately supported by the results?

Yes

Are sufficient details of methods and analysis provided to allow replication by others?

Yes

Reviewer Expertise:

Psychiatric ethics (capacity, surrogate decisions), research ethics, and clinical ethics with specific expertise in medically assisted/induced death policies and practices.

I confirm that I have read this submission and believe that I have an appropriate level of expertise to confirm that it is of an acceptable scientific standard.

# Peer review: Reviewer response for version 1

**DOI:** 10.21956/wellcomeopenres.17922.r40680

**Published:** 2020-10-28

**Authors:** Deborah Ummel

**Affiliations:** 1 Department of Psychoeducation, Université de Sherbrooke, Montréal, QC, Canada

The article that I reviewed aims to address the practical and ethical complexities of medical aid in dying based on the illustrative example of the province of Quebec, Canada, more specifically drawn on nine in-depth interviews with physicians engaged with end of life care.

 This is a welcome article, as empirical data on the matter is indeed needed.

 Regarding study design, the provided description appears insufficient to me. In which paradigmatic position is the inquiry process rooted? As the data analysis mentioned independent analysis by two coders, I would imagine a postpositivst paradigm but this could be clarified.

 I would have been interested on more sociodemographic information regarding the sample (how long have they worked in EOL care, sex, age, working in urban or rural area etc.). What has been the rational to interview more anglophone physicians even if Quebec is primarily a French-speaking province? This aspect should be addressed somewhere in the article. Further, the diversification of the sample given the description provided in the article leaves me doubtful and I would appreciate to read more about this sampling process as well as some justification regarding the sample size.

 Which thematic approach has been used to analyze the data and what steps were involved?

 Why are the quotes followed by physician 1 or physician 2 if there is no table to which to refer to some sociodemographic variables to illustrate those participant? There seems to me that some piece of information is missing.

 The author indicates that nine interviews are not enough to draw generalizable conclusions, which is true, as this is not the goal of qualitative research, even when conducted within a postpositive perspective. There is some consensus regarding the use of transferability in a case like this and this aspect should be addressed in the article. There are numerous authors who have addressed this particular rigor criteria in the qualitative methods literature in the last decades.

 The various lacks in the methods description made me chose partly at the last question, as I would need more information in order to be able to judge of the appropriateness of the proposed conclusions.

Is the work clearly and accurately presented and does it cite the current literature?

Yes

If applicable, is the statistical analysis and its interpretation appropriate?

Not applicable

Are all the source data underlying the results available to ensure full reproducibility?

Partly

Is the study design appropriate and is the work technically sound?

Partly

Are the conclusions drawn adequately supported by the results?

Partly

Are sufficient details of methods and analysis provided to allow replication by others?

Partly

Reviewer Expertise:

palliative and end-of-life care, qualitative research methods.

I confirm that I have read this submission and believe that I have an appropriate level of expertise to confirm that it is of an acceptable scientific standard, however I have significant reservations, as outlined above.
